# Re-thinking of T-tube use in whole liver transplantation: an analysis on the risk of delayed graft function

**DOI:** 10.1007/s13304-022-01267-9

**Published:** 2022-03-24

**Authors:** Riccardo Pravisani, Miriam Isola, Dario Lorenzin, Vittorio Cherchi, Erica Boscolo, Federico Mocchegiani, Giovanni Terrosu, Umberto Baccarani

**Affiliations:** 1 Liver-Kidney Transplantation Unit, Department of Medicine, University of Udine, Udine, Italy; 2 Division of Medical Statistic, Department of Medicine, University of Udine, Udine, Italy; 3 HPB Surgery and Transplantation Unit, Department of Clinical and Experimental Medicine, Polytechnic University of Marche, Ancona, Italy; 4 Dipartimento Di Area Medica, University of Udine, P.Le Kolbe, Via Colugna 50, 33100 Udine, Italy

**Keywords:** T-tube, External biliary drainage, Early allograft dysfunction, Bile acids, Liver transplantation

## Abstract

The liver–gut axis has been identified as crucial mediator of liver regeneration. Thus, the use of a T-tube in liver transplantation (LT), which interrupts the enterohepatic bile circulation, may potentially have a detrimental effect on the early allograft functional recovery. We retrospectively analyzed a cohort of 261 patients transplanted with a whole liver graft, with a duct-to-duct biliary anastomosis, who did not develop any surgical complication within postoperative day 14. Early allograft dysfunction (EAD) was defined according to the criteria of Olthoff et al. (EAD-O), and graded according to the Model for Early Allograft Function (MEAF) score. EAD-O developed in 24.7% of recipients and the median MEAF score was 4.0 [interquartile range 2.9–5.5]. Both MEAF and EAD predicted 90-day post-LT mortality. A T-tube was used in 49.4% of cases (*n* = 129). After a propensity score matching for donor age, cold and warm ischemia time, donor risk index, balance of risk score, Child–Pugh class C, and MELD score, the T-tube group showed a significantly higher prevalence of EAD-O and value of MEAF than the no-T-tube group (EAD-O: 29 [34.1%] vs 16 [19.0%], *p* = 0.027; MEAF 4.5 [3.5–5.7] vs 3.7 [2.9–5.0], *p* = 0.014). In conclusion, T-tube use in LT may be a risk factor for EAD and higher MEAF, irrespective of graft quality and severity of pre-LT liver disease.

**Supplementary Information:**

The online version contains supplementary material available at 10.1007/s13304-022-01267-9.

## Introduction

In liver transplantation (LT), ischemia–reperfusion injury may not only cause a significant impairment of the graft metabolic–biosynthetic function but also result in extensive hepatocytes necrosis, particularly when the graft is burdened by several risk factors such as advanced donor age or graft steatosis [1–3]. In the early post-transplant period, graft functional regeneration is essential, and its failure, clinically diagnosed as early allograft dysfunction (EAD), represents an independent negative prognostic factor for patient and graft survival [1, 2, 4],

The use of a T-tube in LT, an indwelling catheter placed at the level of duct-to-duct anastomosis during biliary reconstruction, has been evaluated so far only in terms of its impact on mechanical biliary complications such as stricture or leakage. However, T-tube interrupts the enterohepatic bile cycle and may induce a persistent local inflammatory reaction, with potential detrimental effects on liver regeneration, as demonstrated in animal models and in liver resection patients [5–11]. However, no data are available in LT setting. Therefore, the aim of the present study was to explore the impact of T-tube use in LT on the risk of EAD, when EAD is defined according to the criteria of Olthoff et al. [2] (EAD-O) and is graded according to the Model for Early Allograft Function (MEAF) score [4]. 

## Methods

### Study population

**Fig. 1:**
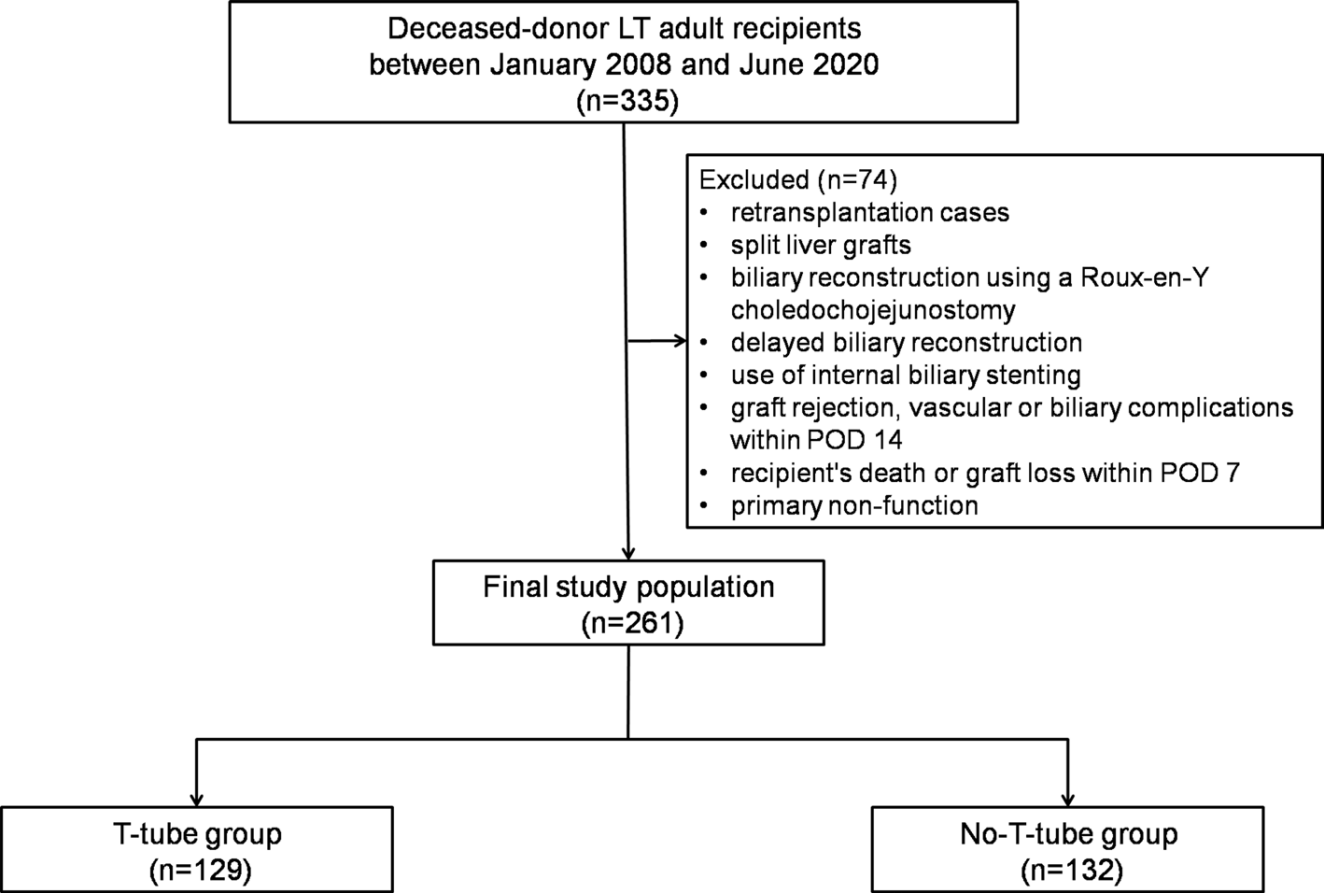
Flowchart detailing the construction of the study cohort with exclusion criteria. *LT* liver transplantation, *POD* postoperative day

This is a retrospective study on a single-center cohort of 335 patients treated with LT at the Liver-Kidney Transplant Unit—Udine University Hospital, between January 2008 and June 2020. Exclusion criteria comprised retransplantation cases, split liver grafts, biliary reconstruction using a Roux-en-Y choledochojejunostomy, delayed biliary reconstruction, use of internal biliary stenting, graft rejection, and vascular or biliary complications within postoperative day (POD) 14. Recipient's death or graft loss within POD 7 was considered an exclusion criteria, because such events precluded a comprehensive calculation of EAD/MEAF. Even primary non-function cases were excluded. The flowchart detailing the construction of the study cohort with exclusion criteria is reported in Fig. 1. The final study population comprised 261 patients.

Demographic and clinical data of the recipients, intraoperative and postoperative outcomes were reviewed from the local electronic database. Liver cirrhosis was diagnosed based on clinical and radiologic criteria and its severity was assessed by Model for end-stage liver disease (MELD) score, Child–Pugh score, and clinical signs of portal hypertension. Demographic and clinical data of the donors were reviewed from the electronic database of the Hospitals where the graft procurement was performed. Liver graft was deemed unacceptable for donation in presence of a biopsy-proven liver graft macro-steatosis over 30–35%. No cases of donation after cardiac death were performed. Graft quality and LT risk profile were retrospectively assessed using the Donor Risk Index [12] (DRI) and the Balance of Risk [13] (BAR) score. Anthropometric donor-recipient matching was assessed in terms of sex mismatch and using the Body Surface Area Index [14] (BSAi).

### T-tube use and postoperative management

During LT procedure, the placement of a T-tube was decided intraoperatively by the senior surgeon without any specific protocol policy. In all recipients, biliary anastomosis was performed end-to-end with continuous 6/0 absorbable suture. In T-tube group, when the posterior side of the anastomosis was completed, a T-tube was placed into the biliary duct through a small incision in the recipient’s bile duct, with the short branch extending into the recipient side and the long branch stenting both the anastomosis and the graft bile duct. Thereafter, the anterior side of the anastomosis was performed. The insertion site of the T-tube was secured with an interrupted suture. The patency of the anastomosis as well as the absence of any leak was always tested with saline infusion trough the T-tube. The T-tube was externalized through the abdominal wall in the right quadrant, fixed to the skin, and connected to a collecting bag. A 2.5-mm rubber tube (Teleflex® Medical, Willy Rüsch GmbH) was routinely used.

Standard post-transplant management for clinical surveillance over postoperative complications was based on daily laboratory tests (full blood count, liver and kidney function) for the first 10 PODs and thereafter as needed according to the clinical course. Hepatic ultrasound with echo color Doppler was routinely performed every day up to POD 7 and thereafter when clinically indicated. When a T-tube was present, it was kept open until a trans-T-tube cholangiography was performed, after POD 7. If no signs of complications were present and serum bilirubin was below 3 mg/dl, the T-tube was closed.

### Primary endpoints

Early allograft dysfunction (EAD) was evaluated according to the MEAF score [4] and according to the definition of Olthoff et al. [2]. Therefore, EAD-O was defined as the presence of one or more of the following previously defined postoperative laboratory analyses reflective of liver injury and function: bilirubin > or = 10 mg/dL on POD 7, international normalized ratio > or = 1.6 on POD 7, and alanine or aspartate aminotransferases > 2000 IU/L within the first 7 days. MEAF [4] was calculated as follows: MEAF = (“score ALT” + “score INR” + “score bilirubin”), where “score ALT” = 3.29/(1 + e ^− 1.9132^(ln(ALT_max.3 days_) −6.1723), “score INR” = 3.29/(1 + e ^− 6.8204^(ln(INR_max.3 days_) − 0.6658), “score bilirubin” = 3.4/(1 + e ^− 1.8005^(ln(bilirubin_day3_) −1.0607).

### Statistical analysis

Categorical variables were expressed by frequencies and percentage, while continuous variables were expressed by median and interquartile range (IQR). Due to imbalances in some baseline characteristics between the study groups, a propensity score analysis was performed. Propensity scores were generated from a multivariable logistic regression model in which T-tube status regressed on baseline with potential pathogenic determinants of early allograft function [3, 12, 13] [donor age, cold ischemia time (CIT), warm ischemia time (WIT), DRI, BAR score, Child–Pugh class C, and MELD score]. The matching method used to generate balanced cohorts was single nearest-neighbor, without replacement [15]. The comparison between T-tube group and no-T-tube group in terms of baseline characteristics as well as EAD-O/MEAF, before and after the propensity score matching, was performed using a Chi-square test for categorical variables and a Mann–Whitney test for continuous variables. Furthermore, the impact of T-tube use on the risk of EAD was also tested in a multivariate model, using a logistic regression for EAD-O and linear regression for MEAF. All analyses were performed using Stata/SE 15.1 (Stata Corp LP, USA). The present study was approved by the local Institutional Review Board.

## Results

The overall study population was characterized by a median age of 59 years [45–69], with a median MELD score of 16 [11–22]. Liver grafts were procured from brain death donors with a median age of 59 years [45–69] and median BMI of 24.8 [23.1–27.2]. The median DRI was 1.7 [1.4–2.0] while the median BAR was 6 [3–10]. The median CIT, WIT, and LT operative time were 7 h 50 min [6 h 20 min–9 h 20 min], 40 min [30 min–50 min] and 6 h 20 min [5 h 30 min–7 h 15 min], respectively. A T-tube was placed at LT in 129 (49.4%) recipients. The prevalence of biliary complications after POD 14 in the study groups was similar (T-tube vs no-T-tube, 11.6% vs 19.7% *p* = 0.073).

The overall median MEAF score was 4.0 [2.9–5.5] and EAD-O was diagnosed in 24.7% (*n* = 63) of cases. Despite the exclusion of cases of patient death or graft loss within POD 7, both MEAF and EAD-O still predicted 90-day post-LT mortality (MEAF: Odds ratio [OR] 1.35, 95% confidence interval [CI] 1.106–1.655, *p* = 0.003; EAD-O: OR 4.654, CI 2.040–10.615, *p* < 0.001). Conversely, 90-day post-LT graft loss (2 cases due to acute hepatic artery thrombosis and 1 case due to uncontrollable graft infection) was not predicted by EAD-O/MEAF.

Recipients with a T-tube showed a higher prevalence of Child–Pugh class C as well as they received grafts from a significantly older donor, with higher DRI and longer CIT (Table 1). After the propensity score matching of the study groups for donor age, Child–Pugh class C, MELD score, DRI, BAR score, CIT, and WIT, the T-tube group still showed a significantly higher prevalence of EAD-0 (T-tube group vs no-T-tube group, 29 [34.1%] vs 16 [19.0%], *p* = 0.027) and a significantly higher value of MEAF (4.5 [3.5–5.7] vs 3.7 [2.9–5.0], *p* = 0.014), as before the propensity score matching. A multivariate analysis confirmed that T-tube use was an independent risk factor for EAD-O and higher MEAF (Table 2).

**Table 1:** Demographic, clinical characteristics of recipients and donors, intraoperative details, and postoperative clinical course

	Full cohort (*n* = 261)			Propensity score matched cohort (*n* = 170)		
	T-tube group (*n* = 129)	No-T-tube group (*n* = 132)	*p*	T-tube group (*n* = 85)	No-T-tube group (*n* = 85)	*p*
Donor and graft characteristics						
Age (years)	64 [52–73]	57 [43–64]	** <0.001**	59 [46–71]	59 [47–66]	0.961
BMI	24.9 [23.1–27.1]	24.8 [23.1–27.5]	0.799	24.5 [22.5–27.4]	25.0 [23.4–28.3]	0.183
Pre-donation ALT (U/L)	26 [15–59]	26 [16–71]	0.687	27 [16–61]	26 [16–44]	0.557
Pre-donation AST (U/L)	29 [20–64]	30 [21–49]	0.990	31 [27–67]	29 [21–48]	0.639
Pre-donation gGT (U/L)	35 [14–70]	33 [15–155]	0.303	36 [14–82]	36 [15–161]	0.381
Pre-donation ALP (U/L)	65 [51–106]	66 [51–95]	0.878	63 [47–126]	65 [51–88]	0.756
Pre-donation sodium (mMol/L)	150 [144–154]	150 [146–156]	0.349	151 [144–154]	150 [146–156]	0.404
Pre-donation lactates (mMol/L)	0.9 [0.6–1.2]	0.7 [0.6–1.2]	0.478	0.9 [0.7–1.1]	0.8 [0.6–1.4]	0.875
ICU-length of stay (days)	4 [2–6]	4 [3–8]	0.175	4 [2–6]	4 [2–7]	0.332
CIT (min)	485 [405–576]	450 [378–540]	**0.033**	482 [405–575]	465 [390–550]	0.218
WIT (min)	40 [32–51]	42 [32–53]	0.403	40 [30–45]	42 [32–50]	0.484
DRI	1.76 [1.51–2.07]	1.52 [1.27–1.96]	**0.008**	1.73 [1.44–1.98]	1.69 [1.37–2.01]	0.777
BAR score	7 [4–10]	5 [3–8]	0.061	7 [4–10]	7 [4–8]	0.620
Donor-recipient sex mismatch (%)	53 (41.1%)	47 (35.6%)	0.363	37 (43.5%)	34 (40%)	0.641
BSAi	0.98 [0.90–1.05]	0.99 [0.90–1.07]	0.293	0.98 [0.90–1.05]	1.0 [0.90–1.06]	0.293
Recipient characteristics						
Age (years)	57 [51–61]	57 [51–62]	0.876	56 [49–62]	57 [51–63]	0.313
Male sex (%)	102 (79.1%)	108 (81.2%)	0.756	65 (74.5%)	71 (83.5%)	0.250
BMI	25.1 [23.0–27.6]	25.1 [22.1–28.1]	0.972	25 [23.0–27.4]	26 [22.7–27.9]	0.485
MELD score	17 [12–24]	15 [11–21]	0.075	16 [11–22]	16 [11–21]	0.423
Child–Pugh class C (%)	60 (47.6%)	42 (33.6%)	**0.024**	39 (45.8%)	32 (37.6%)	0.276
Operative time (min)	385 [330–440]	375 [320–435]	0.570	385 [330–430]	365 [330–430]	0.816
Packed red blood cell transfusion (IU)	4 [2–8]	4 [2–8]	0.630	4 [1–8]	5 [2–8]	0.376
Fresh-frozen plasma transfusion (ml)	1800 [600–3000]	1200 [600–2500]	0.187	1400 [600–3000]	1200 [600–2500]	0.484
EAD-O	46 (35.6%)	17 (13.5%)	** <0.001**	29 (34.1%)	16 (19.0%)	**0.027**
MEAF	4.5 [3.4–5.8]	3.5 [2.7–4.7]	** <0.001**	4.5 [3.5–5.7]	3.7 [2.9–5.0]	**0.014**

Bold values indicate statistical significance

*ALP Alkaline phosphatase, ALT Alanine transaminase, AST Aspartate transaminase, BAR balance of risk, BMI body mass index, BSAi body surface area index, CIT cold ischemia time, DRI donor risk index, EAD-O early allograft dysfunction according to Olthoff et al.’s definition, gGT Gamma-glutamyltransferase, HCC hepatocellular carcinoma, ICU intensive care unit, LT liver transplantation, MELD model for end-stage liver disease, MEAF Model for Early Allograft Function, WIT warm ischemia time
*

**Table 2:** Multivariate model assessing the independent impact of T-tube use on the risk of EAD (EAD-O, MEAF) after groups matching

	EAD-O			MEAF		
	OR	95% confidence interval	*P*	Regression coefficient	95% confidence interval	*p*
Donor age	1.024	1.002–1.046	**0.031**	.013	−.001 to .027	0.082
CIT	1.004	1.001–1.006	**0.011**	.015	.012 to .016	** <0.001**
WIT	1.143	1.019–1.167	** <0.001**	.024	.009 to .040	**0.002**
Child C	.911	.380–2.185	0.836	−.092	−.710 to .526	0.769
MELD	1.031	.977–1.087	0.265	.037	−.002 to .076	0.060
T-tube	1.097	1.003–4.836	**0.002**	.072	.026 to 0.187	**0.002**

Bold values indicate statistical significance

*CIT* cold ischemia time, *EAD-O* early allograft dysfunction according to Olthoff et al.’s definition, *MELD* model for end-stage liver disease, *MEAF* Model for Early Allograft Function, *WIT* warm ischemia time

## Discussion

A T-tube drainage in duct-to-duct anastomosis in deceased-donor LT (DDLT) is traditionally used to monitor the quality and output of bile as a direct marker of graft function, to get an easy radiologic access to the biliary tree, to lower the pressure in the biliary system, to tutor biliary reconstruction, and thus to possibly reduce the incidence of anastomotic stricture [16]. A recent nationwide Italian survey [19] has reported that 25% of Italian LT Centers use it systematically, while 55% use it selectively; another international survey [20] has reported a 33.3% prevalence of T-tube use among LT Centers within the Eurotransplant, Swisstransplant, Scandiatransplant, and British Transplant Society networks. Several systematic review and meta-analysis [16–18, 21, 22] have shown that the overall use of T-tube does not significantly modify the risk of developing either biliary leak or stricture after DDLT. Even in the present study, the prevalence of biliary complications was decreased in the T-tube group, but not at a statistically significant level. Conversely, the impact of T-tube use on the risk of EAD has never been explored so far. In the present study, the recipients with a T-tube had a more severe pre-LT end-stage liver disease (ESLD) and received a lower quality graft, and such features could have explained the increased risk of EAD associated with the T-tube use [3]. However, after propensity score matching for donor age, DRI, BAR score, CIT, WIT, MELD, and Child–Pugh class C, the T-tube use maintained a significant association with both EAD-O and MEAF. The only variable that could not be directly controlled by the propensity score matching was the bile duct size discrepancy between graft and recipient. However, such technical aspect has been mainly identified as a risk factor for biliary complications rather than EAD-O/high-MEAF, and in the present study, the cases with biliary complications within POD 14 were excluded, while the prevalence of biliary complications after POD 14 was comparable between the study groups. Moreover, the study groups showed a comparable BSAi and prevalence of donor-recipient sex mismatch.

In liver resection setting, Otao et al. [11] have shown that patients who underwent major hepatectomy with biliary resection and external biliary drainage not only had significantly lower serum levels of bile acids compared to patients without external biliary drainage, but also had a significantly lower volumetric regeneration of the remnant liver on POD 7. Similar results, although not directly correlated with bile acids deprivation, were reported by Maeda et al. [23] who showed a significantly lower liver remnant regeneration in patients who underwent major hepatectomy with biliary resection and external biliary drainage, compared to those without biliary resection [24]. In experimental models, the bile acids’ (BAs) depletion after major hepatectomy by a bile salt-sequestering resin or by external drainage resulted in reduced liver regrowth [5, 6].

It has been demonstrated that a T-tube externally diverts the majority of bile flow produced by a liver graft, thus interrupting the liver–gut axis [25]. BAs have a selective antimicrobial effect and play crucial regulatory function on the gut microbiota, preventing bacterial overgrowth, controlling the microbiome composition, and modulating its metabolic activity [26, 27]; moreover, they regulate the gut barrier permeability and local immuno-inflammatory response [26, 27]. Thus, the interruption of enterohepatic cycle may result in gut microbiota changes, increased bacterial translocation, toxic intermediate metabolites’ production and absorption, which all have a detrimental effect on liver repair and regeneration [26–28].

Furthermore, in patients with a T-tube, the entire serum BA pool solely derives from hepatic synthesis [25]. Normally, a chronic interruption of the enterohepatic circulation results in a marked compensatory increase in BA synthesis, but after a major hepatectomy or an ischemia–reperfusion injury, this compensatory mechanism may be insufficient [25]. Moreover, the loss of BA metabolism by gut microbiota may further negatively modify the circulating BA pool [25]. Serum BA have been recently identified as important regulatory mediators of liver mass and function [5–7, 26, 27]. While persistent BAs excess has a direct cytotoxic effect on hepatocytes due to increased oxidative stress and cell membrane permeability, physiologic levels and pool of BA are critical mediator of liver regeneration via the farnesoid X receptor signaling [7, 26, 27]. Therefore, the external biliary drainage by a T-tube may potentially deprive the liver graft from an important pro-regenerative trigger.

A persistent proinflammatory state in the biliary tree may impair liver regeneration, as well [9, 10], and T-tube has been associated with an increased risk of postoperative cholangitis or infected bilomas [29, 30], mainly due to T-tube direct bacterial colonization or due increased gut bacteria translocation. In the present study, recipients with biliary complications such as biliary necrosis, leakage, obstruction, or positive bile culture within POD 14 were excluded as potential confounders, and this selection may have at least partially controlled the inflammatory pathogenic mechanism. Even malabsorption due to bile gut deprivation and gut microbiota changes may negatively affect early graft function recovery [26, 28], but unfortunately, no specific data were retrospectively available to assess this potential pathogenic trigger.

If the T-tube use is confirmed as a risk factor for EAD independently from the recipient's condition and graft quality, as it was shown in the present study, a direct potential implication might be a relative contraindication of its use in high-risk LT cases. which may have a greater susceptibility to metabolic/inflammatory detrimental effects of T-tube. Despite a significant heterogeneity among LT centers, an overall trend toward limiting the use of T-tube in LT has been recorded in recent years [19, 20]. Nonetheless, in case of biliary reconstruction with high technical complexity (size discrepancy between graft and recipient bile duct, very small bile ducts) or high risk of bile leakage, the use of a device for biliary tutoring and decompression may still be required [19, 31]. Thus, strategies to reduce the potential functional morbidity of T-tube should be further explored and implemented in clinical practice. Internal biliary stents, protocols of bile replacement, and/or probiotic therapies may be promising options.

The present study shows several limitations: a retrospective modality of data analysis with inherent selection bias associated with T-tube use and limited sample size. Moreover, no specific data on postoperative malabsorption, gut microbiota changes, serum, and biliary BA levels were available. Nonetheless, the present investigation may still have the value of exploring for the first time the pathogenic effect of T-tube beyond purely mechanical complications, clinically assessing how the interruption of the liver–gut axis may negatively affect the early graft functional recovery.

## Supplementary Information

Below is the link to the electronic supplementary material.

Supplementary file1 (TIF 1547 KB)

## Data Availability

The datasets generated during and/or analyzed during the current study are available from the corresponding author on reasonable request.

## Abbreviations

ALP: Alkaline phosphatase
ALT: Alanine transaminase
AST: Aspartate transaminase
BAR: Balance of risk
BMI: Body mass index
BSAi: Body surface area index
CIT: Cold ischemia time
DRI: Donor risk index
DDLT: Deceased-donor liver transplantation
EAD: Early allograft dysfunction
EAD-O: Early allograft dysfunction according to Olthoff et al.’s definition
ESLD: End-stage liver disease
gGT: Gamma-glutamyltransferase
HCC: Hepatocellular carcinoma
IQR: Interquartile range
LT: Liver transplantation
MELD: Model for end-stage liver disease
POD: Postoperative day
MEAF: Model for Early Allograft Function
WIT: Warm ischemia time
