# Supplementary figures and images for: Re-thinking of T-tube use in whole liver transplantation: an analysis on the risk of delayed graft function
Source: Updates Surg. 2022 Mar 24;74(2):571–7. doi: 10.1007/s13304-022-01267-9 (PMC8995289; doi:10.1007/s13304-022-01267-9)

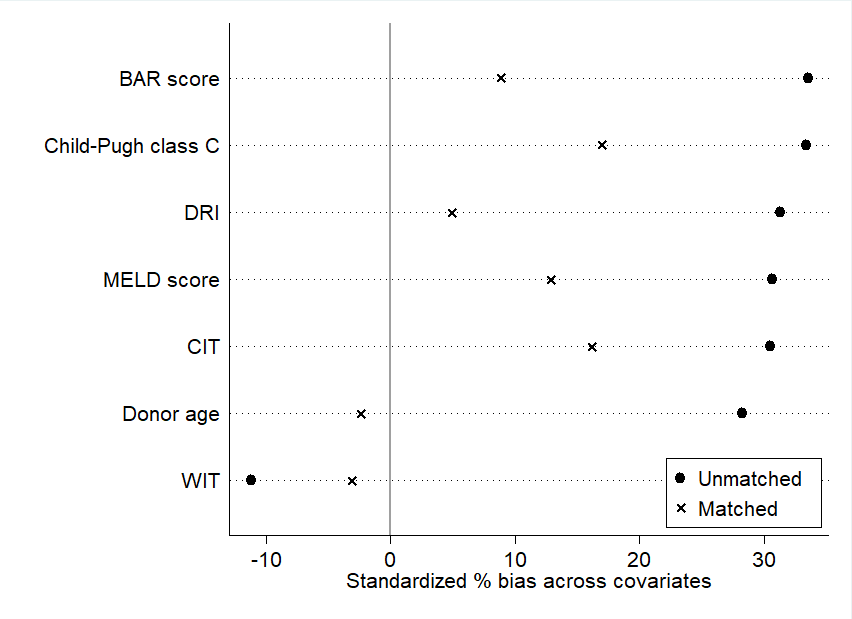

Supplement: Supplementary file 1 — Supplementary file1 (TIF 1547 KB) [file 13304_2022_1267_MOESM1_ESM.tif]
